# Vibration Analysis and Damping Effect of Blade-Hard Coating Composite Structure Based on Base Excitation

**DOI:** 10.3390/ma16155432

**Published:** 2023-08-02

**Authors:** Jiao Wang, Tianyu Guo, Wenyue Liu, Ziwei Wang, Yuehao Zhang

**Affiliations:** 1 School of Electromechanical and Automotive Engineering, Yantai University, Yantai 264005, China; 18253381495@163.com (T.G.); 17861165938@163.com (W.L.); 17865563173@163.com (Z.W.); 2 Engineering Training Center, Yantai University, Yantai 264005, China

**Keywords:** blades, hard coating, damping effect, vibration analysis

## Abstract

Hard coatings are widely employed on blades to enhance impact resistance and mitigate fatigue failure caused by vibration. While previous studies have focused on the dynamic characteristics of beams and plates, research on real blades remains limited. Specifically, there is a lack of investigation into the dynamic characteristics of hard-coated blades under base excitation. In this paper, the finite element model (FEM) of blade-hard coating (BHC) composite structure is established based on finite element methods in which the hard coating (HC) material and the substrate are considered as the isotropic material. Harmonic response analysis is conducted to calculate the resonance amplitude of the composite under base excitation. Numerical simulations and experimental tests are performed to examine the effects of various HC parameters, including energy storage modulus, loss factors, coating thickness, and coating positions, on the dynamic characteristics and vibration reduction of the hard-coated blade composite structures. The results indicate that the difference in natural frequency and modal loss factor of blades increases with higher storage modulus and HC thickness. Moreover, the vibration response of the BHC decreases with higher storage modulus, loss factor, and coating thickness of the HC material. Blades with a complete coating exhibit superior damping effects compared to other coating distributions. These findings are significant for establishing accurate dynamic models of HC composite structures, assessing the effectiveness of HC vibration suppression, and guiding the selection and preparation of HC materials.

## 1. Introduction

Blades play a critical role in aircraft engines and gas turbines. However, they are susceptible to failure caused by vibration fatigue due to exposure to complex vibration patterns. To address this issue, coatings are commonly employed in engineering applications to enhance the wear resistance, corrosion resistance, heat resistance, and other properties of components. The functions and types of coatings vary depending on the specific application field. Soft coatings, such as organic polymer coatings like rubber and viscoelastic materials, have a lower elastic modulus. In contrast, hard coatings encompass metal-based coatings, ceramic-based coatings, and composite coatings that combine metal and ceramic, which possess a relatively higher elastic modulus. Hard coatings offer numerous advantages, including wear resistance, corrosion resistance, fatigue resistance, and radiation resistance, as well as unique dynamic properties and high damping capacity [1]. Consequently, they can be applied to critical components of rotating mechanisms, such as aviation engine blades and gas turbines, which operate under harsh conditions. By increasing the strength and reducing vibration, coatings provide a novel approach to mitigate high-cycle fatigue failure in blades.

HC is commonly utilized as a damper to enhance the dynamic characteristics of structures. It is categorized into three main types: Fe-Cr-Al/Mo metal matrix coatings, MgO + Al_2_O_3_ ceramic matrix coatings, and Sn-Cr-MgO coatings [2,3]. Blackwell et al. [2] conducted research on the damping characteristics of MgO + Al_2_O_3_ coatings. Their findings indicated that when MgO + Al_2_O_3_ coatings were deposited on plates, they exhibited significant damping effects, with the damping behavior relying on the plate’s modes. Ivancic et al. [3] investigated the damping characteristics of fully covered MgO + Al_2_O_3_ coatings on clamped-free titanium plates and identified mode dependence as the dominant characteristic, rather than frequency dependence. A variety of hard coatings, such as CrAlSiN, TiAlN, NiCrAlY, MgO + Al_2_O_3_, and AlCuFeCr/ZrO_2_, have been widely used because they can suppress the vibration of composited structures [4]. Upon discovering the damping properties of HC materials, scholars have conducted extensive research to effectively implement HC damping and vibration reduction. Some researchers have focused on dynamic modeling analyses of hard-coated discs, enhancing their performance by modifying the composition of certain coating elements. Others have investigated methods for identifying and detecting cracks in coatings. However, it is important to note that while these studies have contributed to the understanding and application of HC in vibration reduction, they have primarily focused on simplified structures. Further research is needed to explore the vibration and HC parameters of complex structures, such as blades, under base excitation, to enhance the understanding of their dynamic characteristics and optimize their performance.

The application of hard coatings in the existing literature mostly focuses on simplifying coated discs [5,6,7,8,9,10,11,12,13,14,15], straight blades [16,17,18,19,20,21], plates [22,23,24,25,26], thin shells [27,28,29,30], and disk drums [31]. Yan et al. [5] proposed using virtual layers to simulate changes in coating thickness and analyzed the impact of coating thickness on the natural frequency and forced response of actual disks. Kuang et al. [6] established a finite element reduction model for coated discs, considering the influence of coating thickness changes on the vibration characteristics of the discs. Yan et al. [7] established a reduced-order model of a mistuned disk based on the cyclic symmetry characteristics of the blade disk and studied the effects of coating hardness and coating thickness on the natural frequency, modal loss factor, and forced response of the mistuned disk. Chen et al. [8] used the Rayleigh–Ritz method to calculate the dynamic characteristics of the rotating blisk with NiCrAlY coating on the blades. Considering the effect of coating thickness on the damping effects of the blisk, Gao et al. [9] used NiCoCrAlY + YSZ as an HC material applied to the blade. They used the energy-based finite element method and Newton Raphson method to conduct nonlinear dynamic modeling and forced vibration analysis of the HC disk. Xu et al. [10] presented a novel method based on the mistuning identification technique to detect the blade substrate crack parameters for the hard-coated blisk. Xu et al. [11] investigated a new, equivalent, finite element modeling method based on mistuning identification. Virtual single-layer material blades were used to replace the HC blades of the multilayer structure; for the modal data, the comparison results showed good correspondence between the equivalent model and the actual HC blisk. Xu et al. [12] presented a mistuning identification method for blade cracks in hard-coated blisks based on a modified, component-mode, mistuning, reduced-order model. Results show that, with the increase of a crack length, the mistuning of a crack occurring only in the coating does not increase continuously but decreases firstly and then increases. Gao et al. [13] studied the nonlinear vibration analysis of the integrally bladed, disk-deposited, strain-dependent NiCoCrAlY + YSZ HC on blades and the influence of HC on the dynamics of the blisk. Gao et al. [14] proposed an improved method of passive vibration reduction by the HC damper. They investigated the nonlinear dynamics of the NiCoCrAlY + YSZ HC blisk. Gao et al. [15] developed a passive vibration reduction method by depositing an HC on both sides of the blades. They found that modal loss factors increase as the HC thickens, but its increased gradients reduce gradually. Zhang et al. [16] used CrAlSiN nanocomposite coating for blades to enhance the deformation ability by changing the nitrogen flux and increasing the hardness and toughness of the alloy, thereby reducing the occurrence and propagation of cracks. Zhu et al. [17] proposed using the APS method to study the effect of hard coatings on the natural frequency of cracked blades. They found that hard coatings can delay the initiation of cracks, while hard coatings with larger thickness and elastic modulus can better delay crack propagation. Yuan et al. [18] used experimental and finite element simulation methods to study the addition of TiN coatings on the surface of Ti-6al-4v alloy. The results showed that TiN/Ti multilayer coatings can suppress crack propagation and have good impact resistance. In addition, the HC layer showed strain-dependent and mode-dependent damping characteristics. Zhang et al. [19] studied the vibration characteristics of graphene coating on rotating pre-twisted conical blades. They analyzed the free vibration characteristics of coated blades by changing the geometric shape, pre-twisted angle, rotational speed, etc. of the graphene plate. Chen et al. [20] found that the simulation results of the vibration behavior of the cracked blade were obtained and consistent with the experimental results. Results showed that a NiCrAlY coating was deposited on the blade, and increases in fatigue life were observed under the same condition. Rouleau et al. [21] established a FEM of a blade with a viscoelastic sector, considering the frequency and temperature dependence of viscoelastic materials to study the effects of temperature and rotational speed on damping. Luo et al. [22] proposed a method to reduce the amount of damping material used and conducted experiments to verify it. Tassini et al. [23,24] indicated that stabilized zirconia (YSZ) coatings have amplitude-dependent mechanical properties and nice damping characteristics. Li et al. [25] used the vibrating theory and the finite element iteration method to investigate the dynamic characteristics of the thin plate with HC. Yu et al. [26] theoretically predicted the damping and stiffness of the thin plate coating system and conducted experiments to verify it. Li et al. [27] established a theoretical model of the thin shell structure of composite materials with HC and obtained the natural frequency, modal loss factor, and resonance response values of the thin-walled structure. The theoretical and simulation results are consistent. Zhang et al. [28] studied the nonlinear vibration characteristics of HC cylindrical shells under base excitation, taking into account material damping, equivalent viscous damping, and strain dependence of a hard coating storage modulus and loss factor. Zhang et al. [29] studied the nonlinear vibration characteristics of HC cylindrical shells under base excitation, taking into account material damping, equivalent viscous damping, and strain dependence of a hard coating storage modulus and loss factor. Cui et al. [30] applied hard coatings to aviation hydraulic pipelines to reduce damage caused by vibration. Du et al. [31] applied hard coatings to disk drum coupling structures and found that some coating models had better vibration reduction effects than the entire layer model.

Base excitation is important in the vibration characteristics of testing the structure. Base excitation is widely used since the input energy is stable and excitation is close to the actual stress state of the structure [32,33,34,35]. For example, Araki [32] studied a shaking table as a base excitation to test the response of the structure fixed on the isolator. Pellicano [33] tested the nonlinear vibration characteristics of the cylinder based on base harmonic excitation. Zhou et al. [34] proposed a method for measuring the dynamic response of blades using a single sensor under base excitation and studied the effects of single-frequency and multi-frequency excitation, measurement noise, and selection of measurement points on the response results. Česnik et al. [35] proposed a new method to obtain the stress frequency response function under base excitation using a modal model of unconstrained structures. 

Existing studies primarily concentrate on the vibration characteristics analysis of simplified structures, such as coated discs, straight blades, plates, thin shells, and disk drums. However, limited research has been conducted on the vibration and HC parameters of complex structures, specifically blades, under base excitation. This paper utilizes harmonic response analysis to calculate the resonant amplitude of the composite under base excitation and applies the modal strain energy method to determine the modal loss factor of the composite blade. The study considers the effects of the storage modulus, loss factor, coating thickness, and distribution on the dynamic characteristics of the hard-coated blade composite.

## 2. Vibration Response of the BHC by Base Excitation

**Figure 1:**
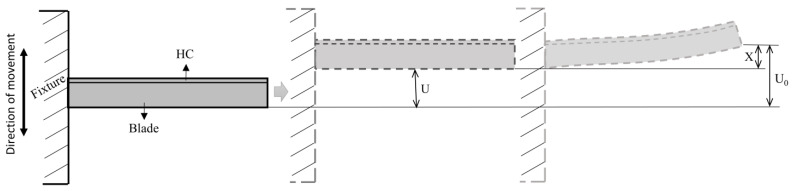
BHC with denoted displacements at the blade tip under base excitation.

The process of basic excitation can be simply described as connecting the BHC composite structural to a rigid platform and using the vibration table to excite the platform foundation. The main characteristic of the basic excitation method is inertial force excitation. Figure 1 shows BHC with denoted displacements at the blade tip under base excitation. Here, U and $\ddot{U}$ denote the displacement and acceleration of the base; X, $\dot{X}$, and $\ddot{X}$ represent the vector of relative displacements, velocity, and acceleration, respectively; and $U_{0}$ and ${\ddot{U}}_{0}$ denote the displacement and acceleration of BHC.

The elastic modulus of the metal base, the damping material layer, and the whole composite structure is expressed by the complex modulus as

(1) $$E^{*}=E(1+i\eta )$$

where * represents the complex number, $E^{*}$ represents the complex modulus of composite structures, and E and η represent the corresponding energy storage modulus and modal loss factor. 

The modal loss factor obtained based on the complex eigenvalue method is

(2) η=Re(ω2)Im(ω2)

where Re(ω2) and Im(ω2) represent the real and imaginary parts of the complex eigenvalues, respectively. 

The relation between $\ddot{U}$ and ${\ddot{U}}_{0}$ can be expressed as

(3) $${\ddot{U}}_{0}=G\ddot{U}+\ddot{X}$$

where G is the influence coefficient vector.

The dynamic equation of the BHC is

(4) $$M_{C}{\ddot{U}}_{0}+C_{C}\dot{X}+K_{C}X=0$$

where $M_{C}$, $C_{C}$, and $K_{C}$ are the mass, damping, complex stiffness matrix, and external force of the BHC, respectively. 

Equation (3) can be used in Equation (4) to obtain

(5) $$M_{C}\ddot{X}+C_{C}\dot{X}+K_{C}X=F$$

where

(6) $$F=-M_{C}G\ddot{U}$$

Equation (6) indicates that the vibration of BHC is generated by base excitation. Assuming that the excitation is harmonic, Equation (5) is converted into the dynamic equation of the BHC under base excitation in the frequency domain [35,36].

(7) $$[K_{C}+i\omega C_{C}-\omega^{2}M_{C}]X=F$$

The corresponding characteristic equation is

(8) $$[K_{C}-\omega_{r}{}^{2}M_{C}]\varphi_{r}=0$$

where $\omega_{r}$ and $\varphi_{r}$ are the r-th order natural circular frequency and regular eigenvectors, respectively.

The obtained modal mode vectors of each order can form a modal mode vector matrix φ. If we pre-multiply Equation (7) by $\varphi^{T}$ and substitute $X=\varphi X_{N}$ into Equation (7), we have

(9) $$\varphi^{T}[K_{C}+i\omega C_{C}-\omega^{2}M_{C}]\varphi X_{N}=F_{N}$$

The corresponding modal coordinate φ base excitation amplitude vector is

(10) $$F_{N}=\varphi^{T}F$$

According to the mode superposition method, the motion equation of BHC in regular coordinates $\varphi_{r}$ is

(11) $$(\varphi_{r}{}^{T}K_{C}\varphi_{r}+i\omega \varphi_{r}{}^{T}C_{C}\varphi_{r}-\omega^{2}\varphi_{r}{}^{T}M_{C}\varphi_{r})x_{Nr}=\varphi_{r}{}^{T}F=f_{Nr}$$

where

(12) $$\varphi_{r}{}^{T}K_{C}\varphi_{r}=\omega_{r}{}^{2}\left[1+i\eta \right],\varphi_{r}{}^{T}M_{C}\varphi_{r}=1$$

According to the proportional damping assumption, Equation (11) can be expressed as

(13) $$\varphi_{r}{}^{T}C_{C}\varphi_{r}=2\zeta_{r}\omega_{r}=\alpha +\beta \omega_{r}^{2}$$

where α and β are proportional constants, and $\zeta_{r}$ is the modal damping ratio corresponding to the r-th order.

Substituting Equations (12) and (13) into (11), we have

(14) $$x_{Nr}^{*}=\frac{f_{Nr}}{\omega_{r}^{2}[1+i\eta ]+i2\omega \zeta_{r}\omega_{r}-\omega^{2}}$$

Then, the steady-state response of the equation in regular coordinates is

(15) $$X_{r}^{*}=x_{Nr}^{*}\varphi_{r}$$

Finally, the vibration response of BHC under base excitation is

(16) $$X^{*}=\sum_{r=1}^{n}X_{r}^{*}$$

## 3. Model of BHC Composite Structure 

### 3.1. Finite Element Modeling Using ANSYS Software

**Figure 2:**
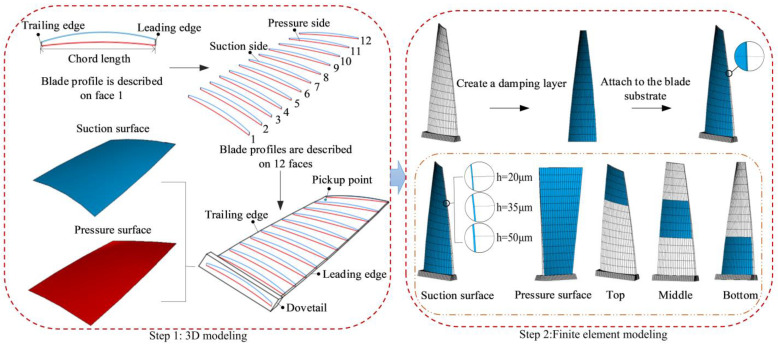
Finite element models of BHC.

The three-dimensional solid model of the blade was obtained by lofting 12 cross-sectional profiles in PTC Creo 4.0 software and then importing ANSYS 17.0 software to obtain the FEM of the blade. During the modeling process, the blade was simplified, ignoring the rounded corners of the actual blade. The complex aerodynamic load was simplified into a simple harmonic force, and the HC material and substrate were simplified into isotropic materials. The specific details are shown in Figure 2. The materials for the blade and HC obtained through experiments are 1Cr11Ni2W2MoV and NiCrAlY, respectively, as detailed in Table 1. The blade substrate can first be mesh, and then HC mesh can be generated along the suction surface to make the nodes and elements of the contact surface of the two layers effectively coupled, fully constraining both sides of the dovetail, as shown in Figure 2. The mesh parameters of the blades and BHC under different working conditions are illustrated in Table 2 in detail. There is only one single variable for each operating condition, and we only want to study the impact of their changes on the vibration and damping effect of hard-coated blades.

**Figure 3:**
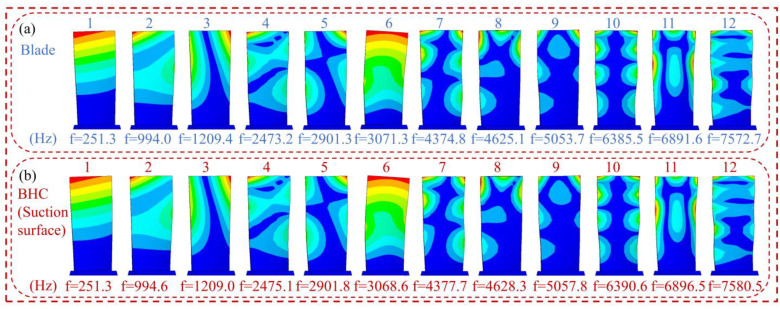
Modal results of the 1–12th order (**a**) blade and (**b**) BHC.

The FEM of BHC with a thickness of 20 μm is shown in Figure 2. The fixed support on both sides of the blade root is used as the boundary condition, which is close to the actual working state, and the Block Lanczos method is used for modal calculation. Their modal shapes and corresponding natural frequencies are listed in Figure 3. As Figure 3 shows, due to the hard thin coating, the natural frequency and the modal shape of the blade change slightly. The first, fifth, eighth, and eleventh-order vibration modes correspond to the bending mode, the torsional mode, the first chord mode, and the “#” mode, respectively. These dangerous vibration modes can easily cause blade fracture and angle loss faults. Therefore, the above-mentioned vibration modes are listed for relevant analysis in this paper.

**Figure 4:**
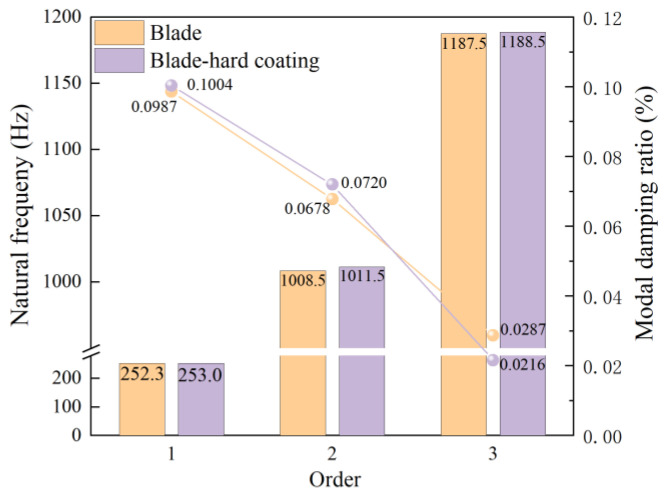
Frequencies and damping ratio of the blade and the BHC by experiment.

From Figure 1, the application direction of acceleration excitation on blades is used to simulate the basic excitation of vibration response. The acceleration excitation forces are 0.5 g, 8 g, 30 g, and 100 g, corresponding to the first, fifth, eighth, and eleventh order, respectively. The vibration pickup point is shown in Figure 2 (Step 1). The full method is used for the vibration response of the blade. Note that150 substeps are applied in the sweep frequency range, and the harmonic load is loaded in steps. According to Equation (17) and Figure 4, Rayleigh damping can be calculated [38]:

(17) $$\{\begin{array}{c} C=\alpha M+\beta K\\ \alpha =2\omega_{1}\omega_{2}\left(\xi_{1}\omega_{2}-\xi_{2}\omega_{1}\right)/\left(\omega_{2}^{2}-\omega_{1}^{2}\right)\\ \beta =2\left(\xi_{2}\omega_{2}-\xi_{1}\omega_{1}\right)/\left(\omega_{2}^{2}-\omega_{1}^{2}\right) \end{array}$$

where $\omega_{1}=2\pi f_{1}$ and $\omega_{2}=2\pi f_{2}$, $f_{1}$, $f_{2}$ and $\xi_{1}$, $\xi_{2}$ are the first two-order natural frequencies and modal damping ratios of the blade measured in the experiment, as shown in Figure 4. The process of obtaining a modal damping ratio through experiments includes using a vibration table with natural frequency as a fixed frequency excitation. After the structural system reaches a steady-state response, the excitation source is cut off. After the excitation is completed, one continues to collect the free vibration attenuation signal of the straight plate blade and uses the envelope method of free vibration attenuation to identify the modal damping ratio of the straight plate blade.

### 3.2. Experimental Verification of Finite Element Modeling Method of BHC

**Figure 5:**
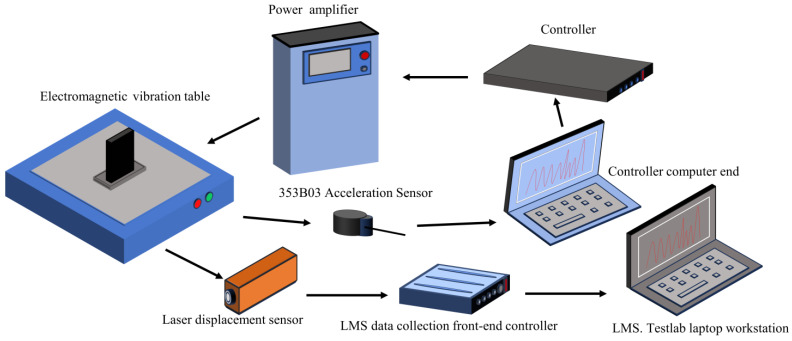
Experimental testing process.

**Figure 6:**
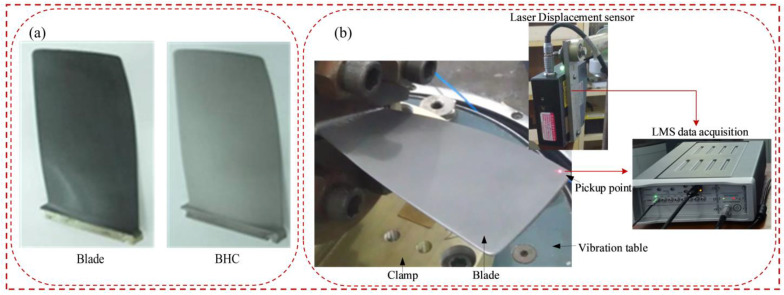
Testing devices and their photo of BHC (**a**) blade and BHC, (**b**) testing.

In this section, the effectiveness of the finite element modeling method for blades and BHC is verified by comparing the natural frequencies and responses of simulations and experiments. The experimental tests include a sweep frequency test and response test under constant frequency excitation. From Figure 5, the blade test piece is installed on the electromagnetic vibration table using a dedicated fixture, fixed and pre-tightened using a torque wrench. The 353B03 acceleration sensor is connected to the electromagnetic vibration table, and it forms closed-loop feedback with the controller computer end, controller, power amplifier, and electromagnetic vibration table. The electromagnetic vibration table is used as the excitation device. A laser displacement sensor is used to obtain the time and frequency domain signals of blade vibration, and the LMS system is used to collect the data. The FEM and the photo in the experiment of BHC are shown in Figure 6.

**Figure 7:**
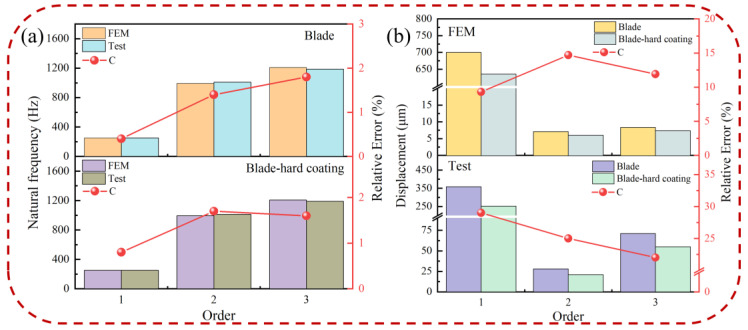
Comparison of the blade and BHC by the finite element method and testing: (**a**) natural frequency and (**b**) displacement.

Based on the test system, the natural frequencies and vibration response of the pure blade and BHC are obtained. Figure 7 compares the natural frequency and displacement response of blades and HBC under the FEM and test. The C($C=(N_{\mathrm{FEM}}-N_{\mathrm{Text}})/N_{\mathrm{Text}}\times 100\%$) in Figure 7a represents the relative error of the natural frequency; the deviation of natural frequencies between the finite element method and the test is less than 2%; and the validity of the FEM and solution method are verified. As shown in Figure 7b, C($C=(R_{\mathrm{Blade}}-R_{\mathrm{BHC}})/R_{\mathrm{Blade}}\times 100\%$) is the relative error of the displacement response. The displacement response of BHC obtained by finite element method and experiment decreased respectively, indicating that HC has a damping effect on blade vibration. The first-order vibration amplitude of the blade is bigger than that of other orders, so the low-order vibration of the blade is more likely to cause the failure of the blade. Since the finite element method is a simplification of the real blade, there is a certain error between the test value and the simulated value. However, it was found that there is a similarity between these two values, so the method of harmonic response analysis of the BHC is feasible.

## 4. Effects of Material Parameters of HC on Vibration Reduction of the Blade

The blade, as an object of vibration reduction, has fixed geometric dimensions and material parameters, which generally do not change. We consider the effect of the material parameters of HC on the dynamic characteristics of the blade. To determine the best coating method, the influence of coating parameters on the vibration characteristics of the blade structure under base excitation is studied. The variable parameters of HC are selected as design parameters, including coating storage modulus, loss factor, thickness, and position. Simulation cases are listed in Table 2.

### 4.1. Case 1: Effect of the Storage Modulus of the HC Material

**Figure 8:**
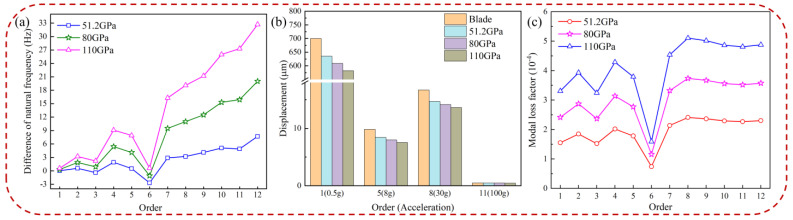
Blade with a different elastic modulus of HC: (**a**) difference of natural frequency, (**b**) displacement of the pickup point, and (**c**) modal loss factor.

Figure 8 describes the effects of the storage modulus on the natural frequency difference, the displacement response of pickup points on the blades, and the modal loss factor, respectively. Figure 8a shows the difference in natural frequency ($K_{\mathrm{BHC}}-K_{\mathrm{Blade}}$). The data in the figure suggest that the difference in natural frequency of the composite structure greatly increases with the increase of the storage modulus of HC. Figure 8b demonstrates that the displacement response at the pickup point on the blade decreases as the storage modulus increases. The influence of the storage modulus on the displacement response is more pronounced in low-order vibrations, effectively reducing blade vibration, while it has little impact on high-order displacement response. Detailed displacement data for blades with different elastic moduli of HC are presented in Table 3. In Figure 8c, it is evident that the modal loss factor increases with the increase in the storage modulus, indicating a more prominent vibration reduction effect with a higher storage modulus. This is because the increased storage modulus enhances the modal loss factor at each order of the BHC, thereby improving the damping characteristics. Furthermore, the natural frequency and modal loss factor exhibit the lowest differences at the sixth order due to the composite mode shape.

**Figure 9:**
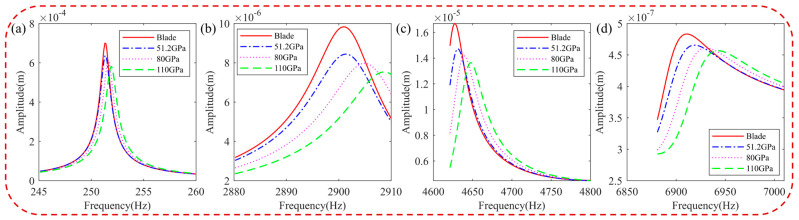
Effect of the storage modulus of the HC material on resonance response of the blade: (**a**) 1st order, (**b**) 5th order, (**c**) 8th order, and (**d**) 11th order.

Figure 9 illustrates the harmonic response of the blades with different storage moduli in the resonance state. It is observed that the frequency of the hard-coated blade shifts and the resonance response amplitude decreases as the storage modulus increases. This reduction in vibration response is attributable to the increased damping characteristics of the BHC resulting from the higher storage modulus.

### 4.2. Case 2: Effect of the Loss Factor of HC Material

**Figure 10:**
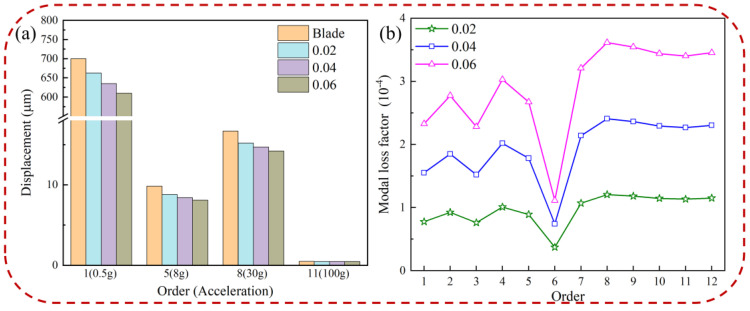
Blade with different loss factors of HC: (**a**) displacement of pickup point and (**b**) modal loss factor.

The influence of the loss factor on displacement response and the modal loss factor of the pickup point on the blade is shown in Figure 10. It can be seen from Figure 10a that the displacement response of the pickup point on the blade decreases with the increase of the loss factor. In addition, the influence of the loss factor on displacement response is that it fluctuates greatly at low order and effectively reduces blade vibration, while it has little influence on high-order displacement response. The detailed displacement data of the blade with different loss factors of HC are shown in Table 4. Figure 10b shows that the modal loss factor increases as the loss factor increases. The increase in the HC loss factor indicates an increase in internal friction of the blade; that is, the stronger the effect of converting vibration energy into thermal energy when the blade receives excitation under base excitation, the more significant the vibration reduction effect. As the loss factor increases, the displacement response and the modal loss factor of the pickup point on the hard-coated blade decreases and increases, respectively, thus indicating that the loss factor can improve the damping characteristics of the blade.

**Figure 11:**
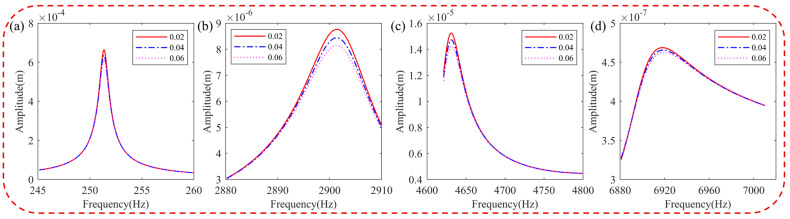
Effect of the loss factor of HC material on the resonance response of the blade: (**a**) 1st order, (**b**) 5th order, (**c**) 8th order, and (**d**) 11th order.

Figure 11 shows the harmonic response analysis of the blade with different loss factors in the resonance state. From the figure, we know that the hard-coated blade’s frequency does not move with the increase of the loss factor, and the resonance response amplitude will be reduced, which indicates that the loss factor cannot change the blade’s natural frequency but can play the effect of vibration damping. This is because the loss factor does not change the stiffness matrix or mass matrix of hard-coated blades, but it increases the damping of hard-coated blades.

### 4.3. Case 3: Effect of the Thickness of HC Material

**Figure 12:**
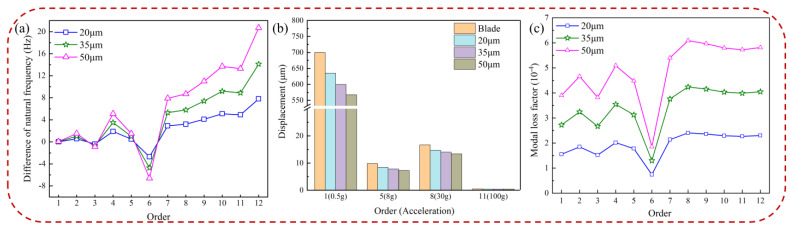
Blade with different thicknesses of HC: (**a**) difference of natural frequencies, (**b**) displacement of pickup point, and (**c**) modal loss factor.

Figure 12 illustrates the effects of HC thickness on the difference in natural frequency, the displacement response of the blade, and the modal loss factor. As shown in Figure 12a,b, the difference in natural frequency and the displacement response of the pickup point increase and decrease, respectively, with an increase in HC thickness. The influence of HC thickness on the displacement response varies greatly in low-order vibrations, effectively reducing blade vibration, while it has minimal impact on the high-order displacement response. Detailed displacement data for blades with different thicknesses of HC are provided in Table 5. Figure 12c shows that the modal loss factor increases with increasing HC thickness, contributing to improved damping characteristics of the coated blade. The increase in the modal loss factor is a result of the higher coating thickness, which enhances the damping effect to some extent.

**Figure 13:**
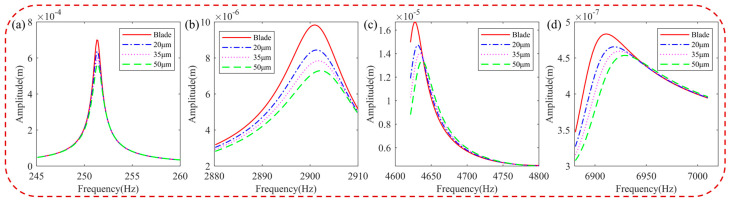
Effect of thickness of HC material on the resonance response of the blade: (**a**) 1st order, (**b**) 5th order, (**c**) 8th order, and (**d**) 11th order.

Figure 13 presents the harmonic response analysis of blades with different HC thicknesses in a resonance state. From the figure, it is evident that the natural frequency of the hard-coated blade remains unchanged at low orders but shifts with an increase in HC thickness at high orders. Moreover, the resonance response amplitude of the blade decreases with increasing HC thickness, indicating that HC thickness can effectively contribute to vibration damping.

### 4.4. Case 4: Effect of the Position of HC

**Figure 14:**
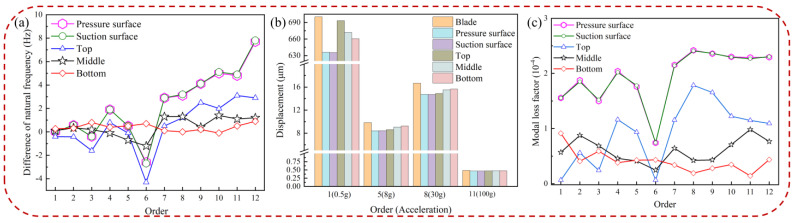
Blade with HC at different positions: (**a**) difference of natural frequencies, (**b**) displacement of pickup point, and (**c**) modal loss factor.

The effects of HC position on the difference in natural frequency, displacement response of the pickup point on the blade, and modal loss factor are depicted in Figure 14. Figure 14a reveals that the difference in natural frequencies between overall coated blades and locally coated blades is greater. In Figure 14b, it is observed that the displacement response of HC added to the pressure surface and suction surface is lower compared to other positions, indicating that the damping characteristics are more pronounced with overall coating rather than local coating. For the specific location of local HC, the displacement response at the bottom of the blade is the smallest in low-order vibrations, while at the top of the blade, it is the smallest in high-order vibrations. Therefore, for the first order of blade vibration, applying HC locally and placing the coating at the bottom yields better vibration reduction effects compared to other positions. For higher-order vibrations of the blade, coating on the top is more effective than coating in the middle or at the bottom. Detailed displacement data for blades with different positions of HC are provided in Table 6. Figure 14c demonstrates that the modal loss factor of rigid coating added to the pressure and suction surfaces is higher than in other locations, and the modal loss factor of the blade with overall coating is greater than that of the locally coated blade, indicating a more pronounced damping effect with overall coating.

**Figure 15:**
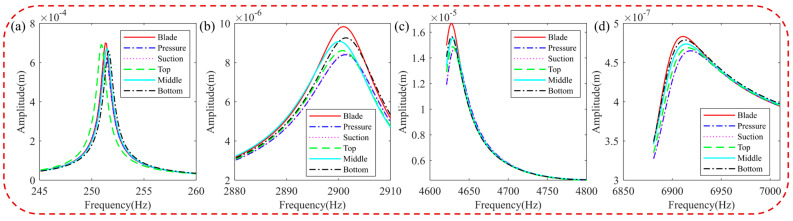
Effect of position of HC on resonance response of the blade: (**a**) 1st order, (**b**) 5th order, (**c**) 8th order, and (**d**) 11th order.

Figure 15 presents the harmonic response analysis of blades with different HC positions in a resonance state. It can be observed that the natural frequency of hard-coated blades shifts with changes in HC position. For lower orders of blade vibration, placing the coating at the bottom is the most effective position for damping, as it experiences maximum stress. If an HC is applied locally, for the lower orders of the blade, placing the coating below is more effective than other positions for damping because the maximum stress of the first vibration mode of the blade is located in the lower part. For higher-order vibrations of the blade, such as the fifth, eighth, and eleventh order, the coating is more effective for the upper part than for the middle and lower parts.

## 5. Discussion

This article verifies the effectiveness of the simulation method for BHC by comparing the natural frequency and displacement response of BHC through experiments and simulations. The findings of this study confirm that the resonance response of the pickup point of the BHC decreases with an increase in the storage modulus, loss factor, and coating thickness of the HC material, aligning with previous research findings [8,27,29]. Similarly, as the HC thickness increases, the natural frequency of the BHC shows an upward trend, consistent with the conclusion from [27]. Furthermore, the shift in the BHC’s natural frequency aligns with the findings of [30].

The study was conducted under static conditions, with certain assumptions made during simulation or experimental settings. These assumptions include disregarding the rounded corners of the actual blade, simplifying complex aerodynamic loads to simple harmonic forces, and treating HC materials and substrates as isotropic materials, which may differ from the actual working conditions. The next step is to address these limitations in order to conduct a comprehensive evaluation of the study.

## 6. Conclusions

This paper utilized harmonic response analysis under base excitation to calculate the resonant amplitude of the composite. The modal strain energy method was employed to determine the modal loss factor of the composite blade. The study analyzed the influence of storage modulus, loss factor, HC thickness, and coating positions on the natural frequency difference, displacement response of pickup points, modal loss factor, and resonance response of blades. The obtained results hold significant implications for establishing a rational dynamic model of HC composite structures and validating the effectiveness of HC vibration suppression. They also provide valuable insights for the selection and preparation of HC materials. The conclusions are as follows:

(1) The HC effectively enhances blade damping, leading to changes in the dynamic characteristics of the blade. The difference in natural frequency and modal loss factor of the blades increases with a higher storage modulus and HC thickness. Additionally, the resonance response and displacement response of the pickup point on the hard-coated blade decrease as the storage modulus, loss factor, and coating thickness of the HC material increase.

(2) The amplitude of a blade with a complete coating is lower compared to that with a partial coating, indicating a more pronounced damping effect of the overall coating. When applying HC locally, for the first-order blade vibration, placing the coating at the bottom yields better vibration reduction results due to the maximum stress concentration at the lower part during the first mode of vibration. Conversely, for higher-order blade vibrations, coating the top exhibits superior effectiveness compared to the middle and bottom positions.

## Figures and Tables

**Table 1:** Material of the blade and HC [37].

Structure	Material	E/GPa	Density/(Kg/m^3^)	Loss Factor	Poisson’s Ratio
Blade	1Cr11Ni2W2MoV	214	7800	0.0007	0.3
HC	NiCrAlY	51.2	2840.7	0.04	0.31

**Table 2:** Number of elements and nodes of the blade and BHC under different working conditions.

Blade Type	Working Conditions			Solid186	
	Cases	Constant Parameters	Vary Parameters	Element Number	Node Number
Blade				3020	14,903
BHC	Case1: Storage modulus Ec/GPa	Hc = 20 μm; δ = 0.04; suction surface	Ec = 51.2, 80, 110	3320	16,210
	Case2: Loss factor δ	Ec = 51.2 GPa; Hc = 20 μm; suction surface	δ = 0.02, 0.04, 0.06		
	Case3: HC thickness Hc/μm	Ec = 51.2 GPa; δ = 0.04; suction surface	Hc = 20, 35, 50		
	Case4: HC position	Ec = 51.2 GPa; Hc = 20 μm; δ = 0.04	Top, Middle, Bottom, Suction surface, Pressure surface	4220 3320	20,270 16,210

**Table 3:** Displacement of the blade with different elastic modulus of HC/μm.

	Orders	1 (0.5 g)	5 (8 g)	8 (30 g)	11 (100 g)
Parameters					
Blade		700.153	9.831	16.69	0.4833
Ec = 51.2 GPa		635.3	8.44	14.7	0.466
Ec = 80 GPa		608.77	7.977	14.17	0.461
Ec = 110 GPa		582.21	7.533	13.646	0.457

**Table 4:** Displacement of the blade with different loss factors of HC/μm.

	Order	1 (0.5 g)	5 (8 g)	8 (30 g)	11 (100 g)
Parameters					
lade		700.153	9.831	16.69	0.4833
δ = 0.02		662.7	8.8	15.2	0.469
δ = 0.04		635.3	8.4	14.7	0.466
δ = 0.06		610.1	8.1	14.2	0.463

**Table 5:** Displacement of the blade with different thicknesses of HC/μm.

	Order	1 (0.5 g)	5 (8 g)	8 (30 g)	11 (100 g)
Parameters					
Blade		700.153	9.831	16.69	0.4833
Hc = 20 μm		635.3	8.44	14.7	0.466
Hc = 35 μm		599.839	7.8415	14.0266	0.459
Hc = 50 μm		567.544	7.2929	13.3855	0.4535

**Table 6:** Effect of different positions of HC on displacement of the blade/μm.

	Order	1 (0.5 g)	5 (8 g)	8 (30 g)	11 (100 g)
Parameters					
Blade		700.153	9.831	16.690	0.483
Pressure surface		635.717	8.400	14.719	0.465
Suction surface		635.300	8.440	14.700	0.466
Top		693.223	8.609	14.875	0.469
Middle		672.014	9.078	15.539	0.476
Bottom		660.502	9.259	15.650	0.478

## Data Availability

The datasets generated and/or analyzed during the current study are available from the corresponding author upon reasonable request.
